# Effects of nandrolone decanoate on femur morphology. Experimental study

**DOI:** 10.1590/ACB360507

**Published:** 2021-06-21

**Authors:** Diogo Benchimol de Souza, Flavia Bittencourt Brasil, Roger Gaspar Marchon, Bruno Félix-Patrício

**Affiliations:** 1 PhD. Urogenital Research Unit - Universidade do Estado do Rio de Janeiro - Rio de Janeiro (RJ), Brazil.; 2 PhD. Laboratory for Research on Translational Histomorphometry - Nature Science Department – Universidade Federal Fluminense - Rio das Ostras (RJ), Brazil; 3 MSc. Urogenital Research Unit - Universidade do Estado do Rio de Janeiro - Rio de Janeiro (RJ), Brazil.; 4 PhD. Laboratory for Research on Translational Histomorphometry - Nature Science Department – Universidade Federal Fluminense - Rio das Ostras (RJ), Brazil.

**Keywords:** Steroids, Femur, Morphology, Rats

## Abstract

**Purpose:**

To evaluate the immediate and late effects of nandrolone on femur morphology of rats.

**Methods:**

Twenty-eight animals with 20 weeks of age were divided into four groups: C28, control animals that were euthanized eight weeks after the experiment started; C40, control animals euthanized 20 weeks after the experiment started; T28, treated animals receiving nandrolone during eight weeks and euthanized immediately after the treatment period; and T40, animals treated during eight weeks and euthanized 12 weeks after the end of the treatment. Treated animals received nandrolone decanoate during eight weeks and control groups received peanut oil by intramuscular injection. After euthanasia, femurs were removed, dissected, weighted and measured by digital pachymeter.

**Results:**

The T40 group presented an increase on distal epiphysis diameter when compared to C40 group. There was no difference between treated and control groups in relation to body and femur absolute weight, relative weight and length of femur. There was also no difference in relation to diameter of proximal epiphysis and diameter of diaphysis among the groups.

**Conclusions:**

Nandrolone decanoate does not produce significant effect on femur, exception on its distal extremity at late period. The effects of such drug may depend on the time after administration.

## Introduction

Nandrolone decanoate is an anabolic-androgenic steroid with several medical applications; however, it is indiscriminately used for fast increase of muscle mass^1^,^2^. Some studies have been performed in order to know its effects in different organs^3^,^4^.

One aspect in which this anabolic has been reckoned is about its possible ability to reverse some bone disturbances, such as osteopenia and those caused by menopause. Some experimental and clinical studies relate that nandrolone decanoate can increase the bone mass both in human and animals and prevent osteopenia^5^,^6^. Such steroid has a positive effect on bone density and mineralization of ovariectomized rats^7^ and restore the carbonate loss in monkeys^8^.

It was also demonstrated that rats that underwent treatment with nandrolone for atrophic fracture nonunion presented bone mass and regeneration without affecting collagen production^9^. Nandrolone helped to increase the mineral density in osteopenic bones of growing rabbits^10^.

On the other hand, some papers report that systemic and local use of nandrolone without physical activity cannot trigger significant changes on some parameters of both bone tissue and muscle mass^11^,^12^ and its effects are sometimes controversial.

According to other authors, nandrolone decanoate can also unleash a variety of changes in several organs, such as cardiac injury, through myocyte hypertrophy, enhancement of matrix type I collagen deposition and hypertension^13^. Besides, it increases the frequency of DNA damage in leukocytes, liver, bone marrow, brain and testicle cells at different tested doses^14^.

Few papers on literature evaluate its effects at different periods after use. This work aimed to assess the immediate and late effects of nandrolone decanoate on femur morphology of adult rats.

## Methods

This project was approved by the local Ethical Committee for care and use of laboratory animals (Protocol No. 755/2016). The experiment was performed at Laboratory for Research on Translational Histomorphometry according to Brazilian legislation for scientific use of the animals.

In order to perform this study, 28 right femurs from 20 weeks old male Wistar rats, weighing 350 to 450 g, were used. The age of animals used are compatible to adult (but not old) animals, to better correlate with humans under anabolic-androgenic steroid abuse. The rats were kept at Universidade Federal Fluminense laboratory, with controlled temperature (25 ± 1 °C) and artificial dark–light cycle (lights on from 7:00 to 19:00). Rats had free access to water and standard food during all experimental period.

Those 28 animals were randomly divided into four groups, each one containing seven animals, as follows: control group – 28 weeks (C28), whose animals were euthanized eight weeks after the beginning of the experiment; control group – 40 weeks (C40), whose animals were euthanized 20 weeks after the beginning of the experiment; treated group – 28 weeks (T28), whose animals were treated during eight weeks and euthanized immediately after the treatment; treated group – 40 weeks (T40), whose animals were treated during eight weeks and euthanized 12 weeks after the end of treatment.

When treated groups reached 20 weeks of age, they underwent chronic use of nandrolone decanoate (Deca Durabolin 50 mg·mL^–1^ Organon, São Paulo, Brazil) at a dose of 10 mg·kg^–1^ of body weight by intramuscular injection, once a week during eight weeks. The control animals received intramuscular injection of vehicle (peanut oil) at the same amount during the same period in order to cause equal stress suffered by the treated animals^3^,^4^.

At the end of experimental period (at 28 or 40 weeks of age), the animals were euthanized with 40 mg·kg^–1^ of thiopental + 10 mg·mL^–1^ of lidocaine hydrochloride 2% (in order to avoid discomfort during injection) mixed in the same syringe. The calculated dose was applied intraperitonially. Immediately after death, femurs were removed, dissected, weighted and measured with a digital pachymeter.

**Figure 1:**
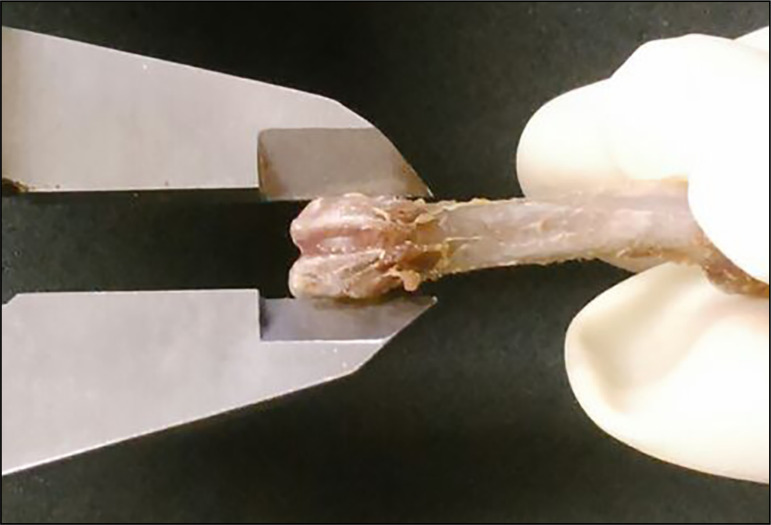
Femur of a rat with its distal epiphysis diameter being measured.

Four femoral measurements were performed according to Lammers *et al*.^15^. For these measurements a digital pachymeter (Starret 799A-6/150, Itu [SP], Brazil) was used. The femur length was determined as the distance (mm) from the most proximal point of the femoral head to the far extremity of the femur. The diameter of femoral diaphysis was determined at the narrowest point of the middle of the femoral diaphysis. The diameter of proximal femur epiphysis was determined from anterior point of femoral head to the tip of the greater trochanter. Finally, the diameter of distal femur epiphysis was considered as the width across the condyles, perpendicular to the length of the femur (Fig. 1).

Also, body weight was measured at the day of euthanasia, as well as absolute and relative weight of the femur. For obtaining the bone weight, the femurs were fully dissected, removing all muscles and tendons. When completely cleaned from any appendix, femur was weighted in an analytical scale (Marte AD500, Sao Paulo [SP], Brazil). Relative weight was calculated by dividing the absolute femur weight by the body weight of each animal.

The means of each parameter were compared by unpaired Student’s t test between groups C28 and T28; C40 and T40; and T28 and T40. In all cases, it was established the significance level of p ≤ 0.05. All analyses were performed by GraphPad Prism 5 software (Graphpad Software, San Diego, USA).

## Results

**Figure 2:**
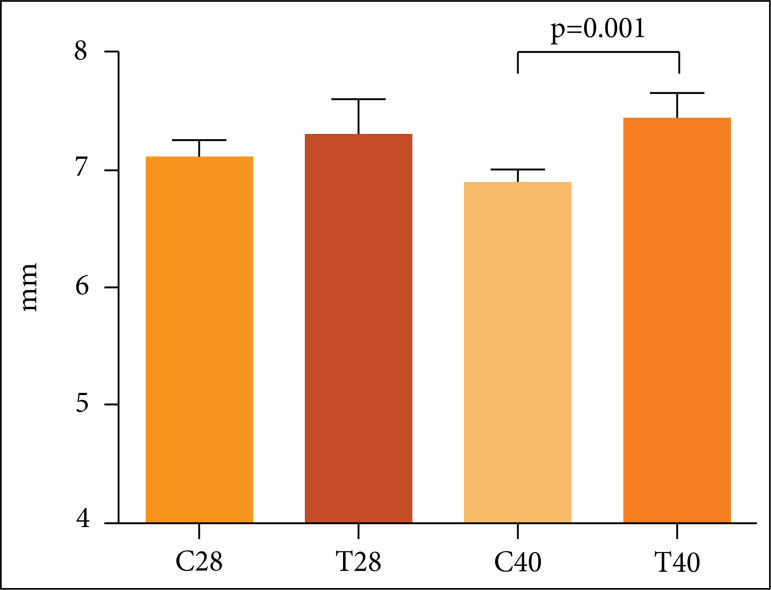
Comparative graphs between distal epiphysis diameter of rats submitted to nandrolone decanoate treatment and controls. Data expressed as mean and SD.

The group T40 presented an increase of 1.7% (p = 0.0013) on diameter of distal epiphysis when compared to C40 group (Fig. 2).

There was no statistic difference between treated and control groups in relation to body and femur absolute weight, relative weight and length of femur. Also, no difference in relation to diameter of proximal epiphysis and diameter of diaphysis. Table 1 shows means ± standard deviation (SD) of all parameters.

**Table 1:** Morphological data from rats submitted to nandrolone decanoate treatment evaluated immediately after the treatment (T28) or lately (T40) and respective control animals (C28 and T28).

Evaluated parameter	C28	T28	p value C28 *vs.* T28	C40	T40	p value C40 *vs*. T40	p value T28 *vs.* T40
Body weight (g)	371.6 ± 59.32	401.2 ± 52.45	0.663	386.0 ± 46.92	421.6 ± 23.00	0.449	0.158
Absolut femur weight (g)	1.140 ± 0.089	1.180 ± 0.085	0.689	1.167 ± 0.103	1.280 ± 0.084	0.096	0.081
Relative femur weight	0.003 ± 0.0003	0.003 ± 0.0004	0.696	0.003 ± 0.0002	0.003 ± 0.0001	0.769	0.918
Femur length (mm)	38.89 ± 1.395	40.13 ± 0.922	0.429	39.85 ± 0.934	40.24 ± 0.638	0.844	0.453
Diameter of proximal epiphysis (mm)	7.766 ± 0.444	8.098 ± 0.228	0.83	8.185 ± 0.263	8.016 ± 0.299	0.638	0.344
Diameter of diaphysis (mm)	4.632 ± 0.246	4.880 ± 0.427	0.273	4.865 ± 0.384	5.034 ± 0.170	0.476	0.388
Diameter of distal epiphysis (mm)	7.092 ± 0.173	6.896 ± 0.107	0.190	7.302 ± 0.280	7.428 ± 0.221	0.001*	0.444

Data are shown as mean ± standard deviation. Means were considered significantly different if p < 0.05.

## Discussion

The results presented in this study show that, among several parameters evaluated, only one was altered in the femur of rats undergone to treatment with nandrolone decanoate. As far as the authors know, this is the first study showing that nandrolone decanoate used during eight weeks can increase the diameter of distal epiphysis in rats.

This experimental model evaluated the use of nandrolone decanoate in animals which were not under physical exercise (except by normal deambulation inside the cage). It is possible to suggest that steroid use without physical activity has low potential to change femur morphology. This can be explained due to the unchanged muscle volume, not inducing drastic bone structural modifications. Camargo Filho *et al*.^11^ demonstrated that there was no difference on soleus muscle fibers diameter, for example, in sedentary animals submitted to steroid administration.

These results are in agreement with Carmo *et al*.^16^, which reported that rats treated with same anabolic drug did not present change on tibia length or soleus muscle hypertrophy. These authors also suggest that the effect of nandrolone decanoate in relation to hypertrophy depends on the type of training performed.

The association between anabolic steroids and intense physical practice did not cause significant increase on muscle mass when compared to animals underwent physical practice without hormonal treatment^17^, reinforcing the effect of physical activity. Similarly, these findings showed that the use of nandrolone decanoate without such activity is not enough to change some parameters, such as body weight, femoral length and weight, diameter of proximal epiphysis and diameter of diaphysis.

Ocarino and Serakides^18^ reported that several factors can regulate bone tissue and physical activity, promoting some changes through direct mechanical force. The application of force generates endogenous signals which influence bone reabsorption and remodeling, besides increasing the connection between osteocytes and its matrix viability^16^.

It has been demonstrated in this study that diameter of distal epiphysis was the only changed parameter. Also, such alteration was not observed in the immediately evaluated group, but only in animals evaluated after 12 weeks of the end of treatment. This suggests that the effects of these hormones take some time to show up and are still occurring even after the end of steroid use.

Kuipers *et al*.^19^ demonstrated that steroids effects are also related to the period in which are administrated. In other study it was reported that administration of nandrolone decanoate exerts effect in ovariectomized rats, increasing the length and femur density^7^. It seems that such steroid has stronger effects on the bone in specific conditions, such as impairment caused by ovariectomy and osteoporosis, in opposite of its use in absence of any pathology.

Future studies comparing the effects of steroids in bones of sedentary versus exercised animals are warranted. Also, in future studies the correlation of bone morphology with muscle hypertrophy are of interest. The study has some limitations that should be pointed. Although the rat is frequently used as an animal model for studying bone morphology, these species do not have comparable weight bearing to humans. Further methods of investigation could be used to depict if there are histological or molecular differences associated with steroids.

## Conclusion

Administration of nandrolone decanoate does not produce short-term effects on femur morphology, but some modifications occur long-term after the end of treatment. The effects of such drugs may take some time to be observed, and are still present even after the end of treatment.
